# Expanding fluorescent base analogue labelling of long RNA by *in vitro* transcription

**DOI:** 10.1016/j.jbc.2025.110825

**Published:** 2025-10-16

**Authors:** Pauline Pfeiffer, Alma F.E. Karlsson, Jesper R. Nilsson, L. Marcus Wilhelmsson

**Affiliations:** 1 Department of Chemistry and Chemical Engineering, Chalmers University of Technology, Gothenburg, Sweden; 2 LanteRNA (Stealth Labels Biotech AB), c/o Chalmers Ventures AB, Gothenburg, Sweden

**Keywords:** RNA, live-cell imaging, *in vitro* transcription, 2CNqA, mRNA labelling

## Abstract

To track RNA inside living cells and to study its natural interactions and mechanisms is vital in RNA research and the development of RNA-based therapeutics. In this work, we show that the fluorescent adenine analogue 2CNqA-triphosphate (2CNqATP) is accepted by the polymerase of a standard *in vitro* transcription, which results in fluorescently labelled long RNA. We demonstrate that the labelling degree can be tuned by the fractional exchange of natural ATP for 2CNqATP in the *in vitro* transcription reaction. Furthermore, in a thorough spectroscopic characterization, we present how the fluorescence lifetime and quantum yield change upon incorporation at different labelling degrees and how this in turn affects overall brightness per RNA strand. After transfecting human cells with the corresponding mRNA, we demonstrate its applicability in live-cell confocal microscopy by simultaneously imaging 2CNqA-labelled RNA and the mCherry reporter protein. We quantified the reporter gene expression using flow cytometry and observed a decrease in mRNA translatability with increasing 2CNqA incorporation. In a benchmark study with Cy5-labelled RNA we reveal that the effect on translatability is similar for both fluorophores. Moreover, we present a general statistical analysis highlighting the importance of considering the effects of unlabelled RNA being translated in cell studies at too low labelling degrees and discuss how this might affect experimental outcome. Despite a decreased protein synthesis, we show that 2CNqATP can be conveniently used to efficiently label and image long RNA, making it a promising fluorescent label in, for example, drug-delivery studies.

RNA is a key molecule performing vital biological functions in all living organisms. From the central dogma of molecular biology, RNA is best known as a coding molecule serving as a messenger (mRNA) in the information flow from DNA to proteins. In addition to this there are non-coding RNA (ncRNA) molecules that perform essential functions inside cells through multiple distinct mechanisms. These ncRNAs are structurally very diverse and can act as catalysts for biochemical reactions (ribozymes) (1)), regulate gene expression (*e.g.,* siRNA and miRNA (2)), modify other RNA molecules (*e.g.,* snoRNA (3)), and even contribute to chromatin remodelling and genome stability (*e.g.,* lncRNA and piRNA (4, 5)). Due to the involvement in these diverse processes, RNA has emerged as both a therapeutic target and a modality for novel treatments, offering new possibilities—particularly for targets previously inaccessible to conventional therapeutics (6).

A recent landmark in establishing RNA as a therapeutic was the approval of mRNA based COVID-19 vaccines (7). Two critical technological advances that individually were the result of decades of research efforts made this development possible, namely: (i) the encapsulation of mRNA into lipid nanoparticles to protect the RNA from degradation and deliver it to cells (8, 9) and (ii) nucleoside base modifications to both reduce immune response and enhance cellular protein synthesis from the delivered mRNA (10, 11). The importance of the latter was highlighted by the 2023 Nobel Prize in Physiology and Medicine to Katalin Karikó and Drew Weissman (NobelPrize.org), and significant research efforts are currently dedicated to identifying new nucleobase analogues that can further improve the therapeutic profile of RNA (12). It is widely recognized that several aspects of RNA biochemistry remain to be untangled for RNA therapeutics to realize their full potential, including the RNA-lipid self-assembly forming lipid nanoparticles, the mechanisms of cellular uptake, and a detailed understanding of the ensuing endosomal pathways and escape (13, 14, 15). Fluorescence-based techniques such as fluorescence microscopy and flow cytometry are imperative research tools in this context, as they enable direct detection of RNA in their native environment. However, canonical nucleobases are virtually non-emissive, and, hence, applying these methods to RNA invariably requires a labelling strategy.

The limitations of most common, bright fluorescent labels stem from the fact that they were not developed specifically for RNA. Their drawbacks include high lipophilicity, bulkiness, and an external (linker-based) attachment. These drawbacks can affect the native structure, interactions with other (bio)molecules, and ultimately the function of RNA itself (16, 17), resulting in data that inaccurately represents its localisation and cellular interactions. Other fluorescence readout methods for visualizing RNA rely on fixating cells (*e.g.,* fluorescence *in situ* hybridization), or require sequence insertion and assembly of multicomponent constructs (*e.g.,* aptamer-based methods) (18), which severely limit their applicability for studying natural dynamic processes. Efforts to identify improved labels for RNA are ongoing and recently, fluorescent base analogues (FBAs) have gained considerable attention as candidates for this purpose (19, 20, 21, 22, 23, 24, 25, 26, 27, 28, 29, 30). In the development of FBAs, organic synthesis, and to an increasing extent computational chemistry advancements, are combined to create a sufficiently bright fluorescent nucleobase capable of maintaining the natural hydrogen bonding and stacking patterns of natural bases to minimize perturbation of the overall structure of the nucleic acid (31, 32, 33, 34, 35). Several FBAs based on both purine and pyrimidine nucleobase scaffolds have been developed and characterized, and their applications for biophysical and biochemical studies are extensive and steadily increasing. For example, our group have employed FBAs in interbase-FRET studies to retrieve structural information on protein-DNA binding (36, 37), to investigate small-molecule binding to DNA and RNA (38, 39), and to study helix-form transitions in DNA and RNA (21, 40). Others have highlighted the use of FBAs in enzymatic reactions to label long (41) and short RNA (42, 43, 44, 45) and to study mRNA:tRNA base-pairing during translation (46). Several FBAs have also been used to label and study therapeutic antisense oligonucleotides inside cells by live-cell imaging (47). To enable live-cell imaging of FBAs incorporated into long RNAs, our group have also developed the synthesis of the ribonucleoside triphosphate version of the cytosine analogue tC^O^, and demonstrated its applicability in *in vitro* reactions to label mRNA (48). By delivering this tC^O^-mRNA to live human cells, we showed that it is still functional in the cellular translation process. In 2022, Wang *et al.* further developed the use of FBAs in RNA labelling when they presented the possibility to metabolically incorporate the cytosine analogue tC into RNA in cells that were engineered for enhanced nucleoside phosphorylation (49). Soon after, we published the spontaneous cellular uptake of our adenine analogue 2CNqA-triphosphate (2CNqATP) and the subsequent incorporation into cellular RNA, which was the first report of metabolic fluorescence labelling of cell-endogenous RNA in non-engineered cells (50).

In this work, we present the incorporation of the fluorescent adenine analogue 2CNqA into RNA by performing a standard *in vitro* transcription (IVT) reaction. By adding different fractions of 2CNqATP to the ATP pool (0–100% of ATP exchanged) in the IVT, we were able to tune the incorporation degree. The resulting RNA was capped and tailed, and its translatability was tested inside living cells. Although protein synthesis was affected, especially at higher labelling degrees, we demonstrate that 2CNqATP can be used to efficiently label and image long RNA. Therefore, together with our previously presented tC^O^TP, which can be used in IVT to label cytosine positions (48), we can now offer higher sequence flexibility for labelling long RNA using base analogues. Additionally, as an adenine analogue, 2CNqA provides the possibility to label the non-coding positions of mRNA, that is, the poly(A)-tail. Furthermore, its favorable properties as a two-photon probe (51) may facilitate future imaging in organoids or tissues. Hence, this work advances the potential applications of FBAs in pharmacokinetic and drug metabolism studies of RNA.

## Results

### Incorporation of 2CNqA into RNA

**Figure 1:**
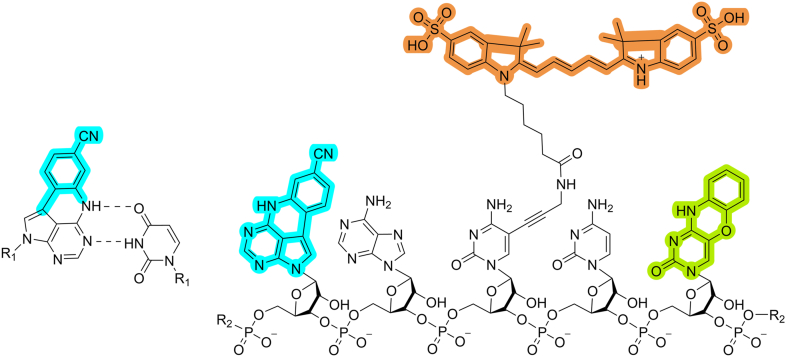
***Left:* Molecular structure of fluorescent base analogue 2CNqA base-paired with uracil.** The chemical modification of 2CNqA compared to the natural adenine base is highlighted in *cyan*. *Dashed lines* indicate hydrogen bonds, and R_1_ and R_2_ represents the RNA sugar phosphate backbone. *Right:* Molecular structure of a representative RNA pentamer with two fluorescent labels of RNA used experimentally in this study – 2CNqA and Cy5 – as well as fluorescent base analogue tC^O^ also discussed in this work. *Cyan*: Fluorescent adenine analogue 2CNqA, *orange*: Cy5 fluorophore attached to cytosine *via* a 5-propargylamino linker, *green*: fluorescent cytosine analogue tC^O^. For comparison, natural A and C are situated between the modifications.

Synthesizing long nucleic acids with defined sequences, like mRNA, typically relies on enzymatic methods such as IVT. In this context, specific intermolecular interactions between the polymerase, cofactors, DNA template, and nucleoside triphosphates are crucial for achieving sequence fidelity and good overall yields, particularly when modified bases are employed. Here, we study whether our fluorescent adenine analogue 2CNqA (Fig. 1, left), which we have previously developed and used in solid-phase synthesis oligonucleotide applications (22, 47, 51), can be incorporated by the commonly used T7 RNA polymerase and compare it to the common label Cy5 (Fig. 1, right).

We added 2CNqATP, that is the nucleoside triphosphate derivative of 2CNqA, in different fractions of the ATP pool to a standard IVT reaction (see Experimental Procedures). Using gel electrophoresis (Fig. S1), we observed that the synthesized RNA, which codes for mCherry, has the expected length (884 nt) independently of 2CNqATP:ATP ratio in the reaction, with the 100% 2CNqATP sample displaying only slightly reduced electrophoretic mobility in the gel.

**Figure 2:**
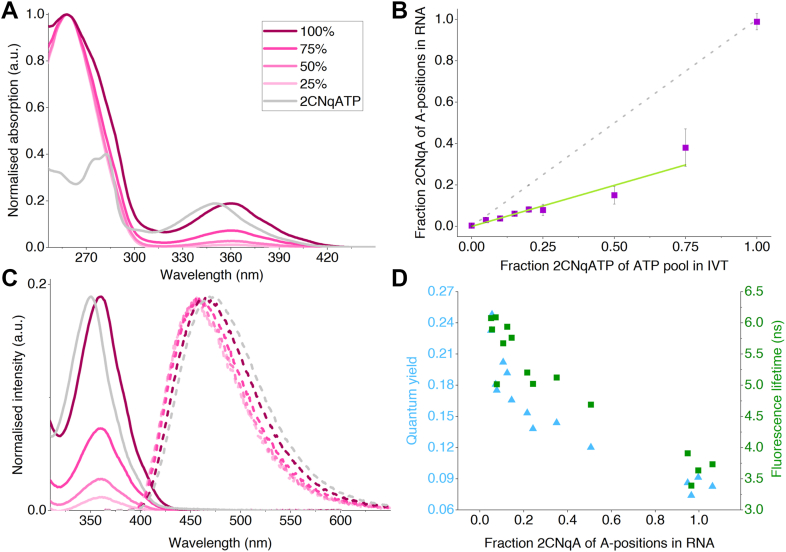
**Photophysical properties of 2CNqA-labelled RNA.***A*, UV-vis spectra normalised to the absorption at 260 nm. The legend denotes the fractions of 2CNqA-triphosphate (2CNqATP) in the ATP pool of the IVT reaction (see Experimental Procedures for details). The absorption spectrum of the 2CNqATP monomer (*grey*) is normalised to the 2CNqA peak at 360 nm in the 100% 2CNqA absorption spectrum for comparison. *B*, 2CNqA incorporation degree, expressed as percentage of adenine positions in the resulting RNA sequence, plotted against the fraction of 2CNqATP of the ATP pool in the IVT reaction. Data is presented as mean ± standard deviation of at least four replicates. A linear fit including the 0%–75% data points resulted in a slope of 0.39. The *dashed line* depicting a 1:1 reactivity is included for comparison. *C*, Zoomed-in UV-vis spectra of 2CNqA-labelled RNA (*solid lines*, compare to A) and the corresponding normalized emission spectra (*dashed lines*, identical color legend as in A). Normalized UV-vis (*solid line*) and emission spectra (*dashed line*) of monomeric 2CNqATP was included for comparison (*grey lines*). *D*, fluorescence quantum yields, and amplitude-weighted fluorescence lifetimes of the individual 2CNqA-labelled RNA samples plotted against the 2CNqA incorporation at A-positions in the RNA, at approximately 20 ng RNA/μl in phosphate buffered saline at room temperature (details in Fig. S4 and Table S1 and S2). 2CNqATP, 2CNqA-triphosphate; IVT, *in vitro* transcription.

After purification using silica spin-columns, the RNA was analyzed spectroscopically to determine purity and 2CNqA content (Fig. 2*A*). The absorption spectra (Fig. 2*A*) clearly show the characteristic RNA peak at 260 nm along with a distinct increasing absorption band at 360 nm attributed to the increased number of incorporated 2CNqA at higher 2CNqATP:ATP ratios. The 360 nm peak, which is consistent with the reported low-energy band of 2CNqA inside RNA (22, 50), is redshifted 10 nm compared to monomeric 2CNqATP in solution (Fig. 2*A*, grey spectrum), also in accord with previous observations (50). Using the absorption values at 260 nm and 360 nm, and the corresponding molar absorptivities of the natural RNA building blocks and 2CNqA, we calculated the fraction of 2CNqA in the synthesized RNA using Beer-Lambert law (Fig. 2*B*, see Experimental Procedures) and the base composition of the resulting RNA prior to poly (A)-tailing (U: 15.4%, G: 29.8%, C: 32.7%, A: 22.2%). Upon plotting the fraction of incorporated 2CNqA (here expressed as fraction of A positions) in the resulting RNA against the fraction of 2CNqATP in the ATP pool in the IVT, we observed a linear behavior for reactions where canonical ATP was present in the IVT reaction (*i.e.,* 0–75% 2CNqATP in Fig. 2*B*). The linear fit of these fractions resulted in a slope of 0.39 representing an apparent reactivity for 2CNqATP:ATP of 1:2.6, which implies that the T7 RNA polymerase has a slight preference for the natural nucleotide over the fluorescent analogue. When ATP was entirely replaced by 2CNqATP in the IVT reaction we observed a 2CNqA incorporation degree of 99% ± 3.9% (Fig. 2*B*). In combination with the observation of the full-length RNA, as shown by gel electrophoresis (Fig. S1), this suggests that it is indeed adenine positions that are replaced by the 2CNqA by the polymerase. Furthermore, as we have not optimized the sequence to reduce the abundance of adenine stretches in the RNA (see distribution in Experimental Procedures), these results show that several 2CNqA can be incorporated adjacent to each other in the RNA strand, which further indicates a good polymerase compatibility of 2CNqA. The reaction yield in terms of the total amount of synthesized RNA decreased with higher 2CNqATP:ATP ratios, which is in accordance with a lower incorporation rate constant for 2CNqATP compared to ATP (Fig. S2). We also observe a non-linear increase in RNA brightness with fraction 2CNqA (Fig. S3), caused by the reduction in quantum yield upon increased incorporation density.

Regarding the photophysical properties of RNA-incorporated 2CNqA, we observe that the emission spectrum of 2CNqA is largely retained (Fig. 2*C*), with a maximum occurring at around 460 nm, which is consistent with the previously reported emission of 2CNqA in RNA (22). The emission spectra of the RNA samples containing different fractions of 2CNqA show a slightly blue-shifted maximum compared to the free monomer. However, at high incorporation degrees, the emission appeared slightly redshifted compared to low and middle incorporation degrees. This subtle shift suggests that the local environment within the RNA, influenced by base stacking, electronic interactions between nearby fluorophores at high labelling degrees as evidenced by circular dichroism (Fig. S5, *vide infra*), structural constraints, and overall conformation, plays a role in modulating the photophysical properties of the analogue. To investigate the photophysical properties in more detail, we determined the fluorescence quantum yield, $\Phi_{F}$, and fluorescence lifetime, $\tau_{F}$, of RNA from four IVT series (25%–100% 2CNqATP in ATP pool; Figs. 2*D* and S4, and Table S1 and S2). This characterization revealed that both fluorescence lifetime and quantum yields decrease as 2CNqA is incorporated into RNA, compared to the monomeric 2CNqATP ($\Phi_{F,TP}=0.48;\;\tau_{F,TP}=9.9\;ns$ (51)), as previously observed for 2CNqA incorporated into oligonucleotides (22, 50). Moreover, the fluorescence lifetime and quantum yield depend on the degree of incorporation, with a decrease of both ($\Phi_{F}$ and $\tau_{F}$) observed with increased 2CNqA incorporation. This coupled decrease indicates a dynamic quenching of the excited state (*vide infra*).

To further investigate the photophysical behavior of 2CNqA incorporated into RNA, we measured circular dichroism (CD) of the RNA with different fractions of 2CNqA (Fig. S5). We observed that the RNA spectral region (200–320 nm) shows an overall unaltered CD, indicating that exchanging adenine for 2CNqA does not change the overall secondary structure of the long RNA. Also, we observed that when all adenines are exchanged to 2CNqA (Fig. S5, 100%), an excitonic CD signal arises over the low energy band. However, for low incorporation degrees, no CD effects were observed in this region.

### Cellular imaging and translation efficiency of 2CNqA-labelled RNA in a human cell line

**Figure 3:**
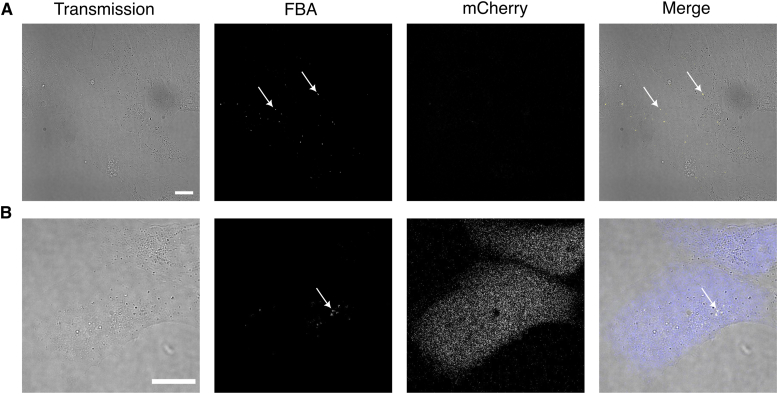
**Live-cell confocal fluorescence microscopy of Huh-7 cells exposed to 2CNqA-labelled mRNA, probing for 2CNqA (*yellow*; λ_ex_ = 405 nm, λ_em_ = 410–490 nm) and mCherry (*blue*; λ_ex_ = 560 nm, λ_em_ = 589–750 nm).** The scale bar represents 20 μm. *A*, 30 min after exposure to 2.1%-labelled 2CNqA-RNA. *B*, Five hours after exposure to 1.1%-labelled 2CNqA-RNA (images from 30 min after exposure in Fig. S7).

To investigate the performance of 2CNqA-labelled mRNA inside live cells, we first installed a Cap0 structure and poly(A) tail in the 5′ and 3′ end of the *in vitro* transcribed RNA, respectively. The resulting mRNA was then formulated using lipofectamine and added to WT Huh-7 cells while monitoring the uptake using confocal fluorescence microscopy. We observed that 2CNqA-RNA with an incorporation degree as low as 1%–2% could successfully be detected as bright spots inside the cells already after 30 min exposure, using the microscope’s 405 nm laser line (Fig. 3). The fluorescent mCherry reporter gene was selected and implemented because of its spectral separation from the 2CNqA emission, which could facilitate convenient mRNA detection independently from its translation product. To investigate if the 2CNqA-labelled mRNA could be imaged alongside the mCherry protein, we exposed cells to 2CNqA-RNA with a labelling degree of 1.1% and captured images in a time-lapse manner while probing for both 2CNqA and mCherry. After 5 h exposure, we observed diffuse mCherry fluorescence across the cytosol and nuclei of the cells (Fig. 3*B*; microscopy images of lipofection without RNA and with unlabelled RNA is found in Fig. S6), consistent with the expected localization of the mCherry protein. These results show that the 2CNqA-labelled mRNA can indeed be processed by the ribosome and translated into a functional fluorescent protein and that the labelled mRNA and the fluorescent protein can be tracked simultaneously using two separate channels in the microscope (Fig. 3*B*).

**Figure 4:**
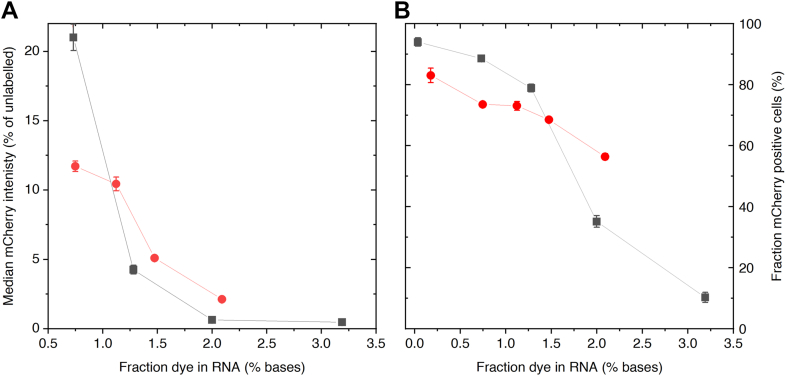
**Flow cytometry analysis of Huh-7 cells exposed to mRNA labelled with 2CNqA (*red circles*) and Cy5 (*black squares*) at different incorporation degrees.** Cells were exposed to lipofectamine-formulated mRNA for 24 h and all events were gated for single living cells for analysis. Data is presented as mean ± S.D. of three independent replicates, each in technical duplicates. Intensity histograms are presented in Fig. S8 and S11. *A*, median mCherry intensity normalized to the corresponding intensity from cells exposed to unlabeled mRNA. *B*, fraction mCherry-positive cells.

To quantify the *in cellulo* translatability of 2CNqA-labelled mRNA we used flow cytometry. Since the mCherry signal from RNA with a 2CNqA incorporation degree of 2.1% appeared low in the microscopy images (data not shown) but was readily detectable using RNA containing 1.1% 2CNqA (Fig. 3*B*), we decided to further investigate the translation using a series of mRNAs covering this incorporation range (0.8–2% 2CNqA in RNA). The observed median fluorescence intensity from the single living cells population shows that 2CNqA causes a decrease in the protein synthesis (Figs. 4*A* and S8), with the mCherry intensity dropping by a factor of ten for the lowest investigated incorporation degree (0.8%), compared to the mCherry signal from the unlabelled RNA. This implies that the presence of 2CNqA affects, yet does not completely inhibit, the translation of the mRNA. By introducing an intensity threshold based on the signal from unexposed cells (representing the autofluorescence level, Fig. S8), the fraction of single living cells capable of synthesizing mCherry (mCherry positive) was determined (Fig. 4*B*). From this analysis, we observe that the overall median mCherry intensity has a stronger dependence on incorporation degree than the corresponding fraction of mCherry positive cells.

Overall, the 2CNqA-labelled mRNA could be detected inside cells using a standard confocal fluorescence microscope setup, even at low fluorophore incorporation degrees. However, even at such low levels of 2CNqA incorporated into the mRNA, we observe a significant effect on translatability. For mRNA with very low 2CNqA incorporation (or any stochastically incorporated base), there is a statistical probability that a non-negligible fraction of the RNA strands is unlabelled. To investigate if the mCherry fluorescence signal in our results originated from such an unlabelled fraction, binomial statistics was applied to calculate the probability of having unlabelled strands as a function of number of nucleotides in the sequence, number of adenine sites per strand, and incorporation degree (see Experimental Procedures and Fig. S9). This analysis showed that at the lowest 2CNqA incorporation degree (0.8%), the probability of a strand being unlabelled was 0.08%, suggesting that the protein expression we observe in Figures 3 and 4 must originate from mRNAs containing at least one 2CNqA.

### Benchmark against Cy5-RNA

To compare our results to a fluorophore commonly used for RNA labelling, we performed IVT reactions using a commercially available Cy5-CTP, where the Cy5-dye is attached to a cytosine triphosphate *via* a 5-propargylamino linker (Fig. 1). Following incorporation by IVT, this results in an RNA which, unlike 2CNqA-labelling, is labelled outside of the base stack. We used the same DNA template as for the 2CNqA-IVTs and prepared a series of incorporation degrees by varying the Cy5-CTP fraction in the CTP-pool (Fig. S10*A*). After purification, the Cy5-RNA was analyzed by gel electrophoresis and UV-vis, and the incorporation degree was calculated (Fig. S10). The resulting plot of incorporated Cy5-C as a function of Cy5-CTP fractions in the CTP-pool of the IVT reaction was fitted to a linear equation with a resulting slope of 0.23, that is an apparent reactivity of 1:4.3, which indicates a lower incorporation preference for Cy5-CTP compared to that of 2CNqA (0.39; 1:2.6, *vide supra*) (Fig. S10*B*). However, it should be noted that the CTP-pool concentration was lower in these IVT reactions compared to the ATP-pool concentration in the 2CNqA labelling reactions (see Experimental Procedures). This analysis suggests that the T7 RNA polymerase accepts the smaller and more native-like 2CNqA better than Cy5. After capping and tailing the Cy5-RNA, we analyzed its translatability by flow cytometry in the same way as for 2CNqA-mRNA (Figs. 4 and S11). We observed that the translation efficiency was negatively affected by the fluorophore incorporation to a similar extent as it was for 2CNqA, and that the fraction of mCherry positive cells following Cy5-labelled mRNA exposure follows the same trend as for 2CNqA-labelled mRNA. A statistical analysis to evaluate the probability of having unlabelled RNA strands contributing to protein expression was performed with respect to cytosine positions in the sequence (Fig. S9, compare adenine positions for 2CNqA, *vide supra*). This showed that the probability that a strand is unlabelled is 0.2% at the lowest incorporation degree of the Cy5-RNA series (0.7% Cy5), which implies that the mCherry signal observed here likely does originate from Cy5-labelled RNA. However, it is clear that for typical fluorescent reporter RNAs with lower incorporation degrees (*ca.* 1000 nt; <0.3% incorporation), which occasionally are provided by commercial Cy5-mRNA suppliers, a considerable amount of unlabeled RNA strands is statistically likely to be present (Fig. S9).

## Discussion

In this work, we investigated the applicability of the fluorescent adenosine triphosphate analogue 2CNqATP as a building block for IVT to label long RNA and studied the translation compatibility of 2CNqA-labelled mRNAs in live cells. We also present a detailed spectroscopic investigation of the RNA, where we determined the incorporation degree of 2CNqA, as well as the fluorescence quantum yields ($\Phi_{F}$) and fluorescence lifetimes ($\tau_{F}$) at these varying RNA labelling degrees – characteristics that are important for designing experiments involving fluorescence-based detection, such as fluorescence microscopy. We also synthesized and evaluated a series of Cy5-labelled mRNA, focusing specifically on benchmarking the transcription and translation. Prior to this work, 2CNqA has been thoroughly characterized as a nucleoside as well as a triphosphate. It has also been utilized in metabolic labelling of RNA, incorporated into oligonucleotides, and shown to be a versatile fluorescent label in flow cytometry and fluorescence live-cell imaging in single- and two-photon mode (22, 47, 50, 51). With these properties, we reasoned that 2CNqA may be a suitable candidate for labelling long RNA for downstream applications in live cells. We added the nucleoside triphosphate derivative 2CNqATP, previously synthesised in our lab, in different 2CNqATP:ATP ratios to standard T7 RNA polymerase IVT reactions and found that the polymerase produces fluorescent RNA of the expected size. Comparing to the other samples, the slight shift in electrophoretic mobility observed for RNA synthesised with complete replacement of ATP to 2CNqATP (Fig. S1), is likely not an effect of change in transcript size, but rather a slight change in hydrophobicity and/or secondary structure, caused by the high 2CNqA incorporation. Since 2CNqA has an additional, lower-energy absorption band around 360 nm compared to the canonical bases, the degree of 2CNqA incorporation can be determined by analyzing the UV-vis spectra of the purified transcripts. By relating the incorporation degree to the added fraction of 2CNqATP in the reactions, it was seen that although the T7 RNA polymerase accepted 2CNqATP well, canonical ATP is favored by a factor of 2.6 (Fig. 2*B*), which results in reduced reaction yields for IVT reactions at high 2CNqATP:ATP ratios (>25% of ATP replaced, Fig. S2). This is in contrast to our previously reported cytidine triphosphate analogue tC^O^TP (Fig. 1, right), which was incorporated equally well as the canonical CTP by T7 RNA polymerase (48). It should be noted, however, that compared to Cy5-CTP, which was disfavoured by a factor of 4.3 compared to natural CTP (Fig. S10), 2CNqATP offers a more beneficial labelling economy. While all three fluorescent nucleosides (Cy5-C, 2CNqA, tC^O^; Fig. 1, right) have intact Watson-Crick hydrogen-bonding pattern with the natural base on the opposite strand, the positioning of the fluorophore moiety in the RNA strand is substantially different. Cy5-C is carrying the fluorophore *via* a flexible covalent linker, while tC^O^ and 2CNqA are nucleobase analogues, for which the aromatic ring-system itself has been extended, and therefore the bases themselves are the fluorophores (Fig. 1, right). Hence, solely considering the bulkiness of the labels (Cy5-CTP > 2CNqATP > tC^O^TP), the results in this work point to an overall decrease in polymerase processivity with increasing size of the fluorescent nucleoside triphosphate, suggesting that steric clashes between the polymerase active site and the modified ribonucleoside triphosphates (XTP) plays a role. Together with the previous observation that tC^O^TP barely displays any change in incorporation compared to natural CTP indicates that there are other parameters in addition to size determining substrate suitability. Such parameters, which are different for Cy5-CTP, 2CNqATP, and tC^O^TP, include pi-stacking properties and hydrophobicity. To unveil the details of how these structural characteristics contribute to RNA polymerase processivity of XTP fluorescent labels, a comprehensive structure-activity relationship (SAR) study is required. One could also imagine furthering this understanding by investigating the enzymatic incorporation of 2CNqATP and other XTPs using various polymerases, for example bacteriophage polymerases T3 and SP6 (52, 53), or polymerases from the eucaryotic system yeast (54). A more systematic, yet tedious, approach would be to implement purpose-made RNA polymerase mutant libraries.

Regarding the photophysical properties of 2CNqA inside RNA, we observed that the emission spectrum is only slightly shifted, and with an increasing incorporation degree, both fluorescence lifetime and quantum yield decrease (Fig. 2, *C* and *D*). A similar trend was previously observed for the fluorescent cytosine analogue tC^O^ in RNA (48), and we attribute this effect to an overall increase of proximal fluorescent base analogues in the RNA. The coupled decrease in lifetime and quantum yield (Fig. 2*D*) moreover indicates a dynamic quenching of the excited state, possibly *via* oxygen quenching (55) due to an increased homo-energy transfer between increasingly proximal 2CNqA bases. Another consequence of an augmented incorporation degree is the probability of having two or several 2CNqAs as direct neighbors in the RNA strand. As an effect of the significantly lower reactivity of 2CNqATP compared to ATP, RNA produced with as much as 75% 2CNqATP (25% ATP) does not contain 2CNqA in more than ca 35% of the A-positions (Fig. 2*B*) and, consequently, only a low amount of direct 2CNqA-2CNqA neighbours. On the other hand, the sample with 100% of exchanged ATP will understandably contain 2CNqA at all A-positions, resulting in several direct 2CNqA-2CNqA neighbors (see adenine neighbor distributions in Experimental Procedures). This effect can clearly be observed in the CD (Fig. S5) where an exciton effect appears in the low-wavelength region. This CD effect is positive at lower energies and has the expected cross-over at the center of the 2CNqA S_0_-S_1_ absorption band (360 nm). At wavelengths just below 360 nm a negative band corresponding to the positive one above 360 nm is expected for exciton CD. However, in that region we observe a signal which is only slightly below zero (Fig. S5). This is most likely due to minor involvement of overlapping absorptions of 2CNqA in this region and overall small CD signals obstructing reliable interpretation of the data. The observed exciton effect suggests an electronic interaction between adjacent 2CNqA fluorophores resulting in new electronic ground and excited states of the 2CNqA fluorophore. These new states influence the overall photophysical behavior since they give rise to new chromophore species having photophysics that differ from those of an isolated 2CNqA inside RNA. Hence, the lower quantum yield and shorter lifetimes at higher labelling densities (Fig. 2*D*) are likely due to a combination of dynamic quenching by oxygen and electronic coupling between proximal 2CNqAs within the RNA.

A crucial parameter for many fluorescence-based applications is the detectability of RNA, which is dictated by the brightness (*i.e.,* the molar absorptivity × fluorescence quantum yield) per strand. For 2CNqA in this context, the effect of increasing the incorporation degree by using more 2CNqATP in the IVT, which increases incorporation (and thus the molar absorptivity), is counteracted by the herein observed decrease in fluorescence quantum yield, causing a non-linear relation between incorporation degree and brightness (Fig. S3). This non-linearity is important to consider when designing 2CNqA labelling reactions for applications where brightness is critical, for example for fluorescence-based quantification purposes.

To render RNA translation competent, we capped and tailed the *in vitro* transcribed RNA. Opting for a post-transcriptional tailing reaction, rather than encoding the poly(A) tail in the DNA template, was motivated by a desire to focus the study on labelling the protein-coding region of the RNA by avoiding that 2CNqA, being an adenine analogue, localizes to the poly(A) tail during transcription. Since we found during our investigation that increased incorporation of 2CNqA into RNA reduces the translation efficiency we opted here for a low labelling density which is even outside the range of the labelling density characterized in Figure 2. We thereafter transfected human hepatocytes (Huh-7) with mRNA using a well-established lipofection protocol and imaged the 2CNqA-labelled mRNA inside cells using the standard 405 nm laser line of the confocal microscope. Despite the absorption maximum of 2CNqA being around 360 nm (Fig. 2*A*), excitation into the tail of the low-energy band still allowed for localization of the RNA even with a comparably low incorporation degree (1.1% 2CNqA, Fig. 3*B*). The signal from the RNA, similarly to what was previously observed for tC^O^-labelled RNA (48), appears as bright spots inside the cells. RNA delivered to cells *via* lipofection generally follows the endocytic pathway (56), and, hence, the bright spots that we observe are most likely vesicles of the endocytic uptake pathway containing the labelled mRNA molecules. By using a DNA template that was designed to code for the red-emitting mCherry protein, and that furthermore contained the necessary features for efficient translation in eukaryotic cells (UTRs, Kozak sequence, and stop codon), we could directly investigate the translatability of our 2CNqA-labelled mRNA *via* the mCherry fluorescence, without spectral bleed-through from 2CNqA. Upon exposing cells to 2CNqA-mRNA with low incorporation degrees, we were able to simultaneously track both the mRNA and mCherry reporter protein using two separate channels in the microscope (Fig. 3*B*). However, the images clearly showed that the 2CNqA labelling had a negative effect on RNA translation. To further investigate this effect, we analyzed Huh-7 cells using flow cytometry, which revealed that the protein production is indeed affected in an incorporation degree-dependent manner (Fig. 4). While the total mCherry signal exhibits a strong dependence on 2CNqA incorporation, the number of cells translating mCherry is affected comparably little (Fig. 4). This could be an overlapping effect of altered endosomal escape from the lipoplexes inside the cells, differences in the interactions between the labelled RNA and the ribosome, or even a misreading of the analogue as another base than adenine, which could result in a non-fluorescent protein. Regarding the recognition of 2CNqA as an adenine analogue, previous studies have shown that 2CNqA, when incorporated at an adenine position, maintains both the Watson-Crick base-pairing and the A-form helical structure of RNA, although having a slightly stabilizing effect on RNA duplexes (22). This suggests that the base-pairing interactions of the 2CNqA-labelled mRNA with tRNA inside the ribosome likely are unaffected, but that the extended and more hydrophobic ring system of 2CNqA, compared to adenine, could alter and/or sterically hinder the interactions at the site of translation in the ribosome. We observe a similar decrease in translation and number of mCherry-expressing cells when labelling the RNA with Cy5, which, considering the substantial size of Cy5, further supports this notion (Fig. 4). On the other hand, considering that the tricyclic cytosine tC^O^, which is extended to a comparable extent as 2CNqA (Fig. 1, right), but has been reported to have a significantly smaller perturbing effect on the translation (48) compared to what we herein report for 2CNqA, the compromised translatability of 2CNqA-mRNA is likely not only a consequence of size or steric hindrance. Instead, since it has previously been established that 2CNqA increases duplex stability (22), we suggest that it may induce changes in the secondary and possibly tertiary structure of the mRNA. This could in turn affect its ability to form the RNA-protein complexes required for efficient translation initiation, including the scanning of a ribosome subunit along the mRNA and recognition of the first AUG as start codon (57), as well as the mRNA unwinding by the ribosome during elongation of the amino acid chain (58). Such changes of mRNA structure may occur also for other RNA labels, including the Cy5 and tC^O^ discussed here, which makes it an important parameter to consider for all fluorescence labelling approaches involving mRNA.

In conclusion, we present the bright fluorescent base analogue 2CNqA which, as a triphosphate derivative, can be incorporated into RNA using a standard T7 RNA polymerase *in vitro* transcription reaction. We found that translation of the corresponding 2CNqA-labelled mRNA in human cells decreases with increasing incorporation degree, yet, at a suitably low incorporation degree, it can be used to simultaneously track both the mRNA and its protein product. The fact that 2CNqA is an adenine analogue offers improved flexibility in terms of what type of positions that can be labelled in long RNAs, adding to the previously developed cytosine analogue tC^O^ (48) (Fig. 1, right). We have previously shown that 2CNqA-labelled oligonucleotides are compatible with two-photon live-cell microscopy (51) which, together with the findings from this study, allows for using 2CNqA to also study long RNA in two-photon mode. The longer wavelengths used in two-photon microscopy (typically 700–800 nm for these types of fluorophores), provide improved tissue penetration and reduce phototoxicity, which could make 2CNqA a promising label for studying mRNA in, for example, organoids or tissues. In general, the possibility of labelling long RNAs at different base-positions enables detailed studies of RNA delivery processes using live-cell imaging with downstream image analysis. Apart from pharmacokinetic and drug metabolism studies of RNA therapeutics, potential research applications where 2CNqA may be utilized include investigations on RNA turnover or tracking the fate of different RNA molecules inside cells. For example, we have ongoing studies in our lab assessing the possibility of using 2CNqATP to label the poly(A) tail of mRNA, which is an interesting strategy for labelling mRNA outside the coding region. Together with the herein observed difference in fluorescence lifetime of incorporated *vs.* monomeric 2CNqA, this may facilitate the study of kinetics of mRNA turnover by detecting the change in fluorescence lifetime as the poly(A)-tail degrades.

## Experimental procedures

### DNA template for *in vitro* transcription

The DNA template was designed by LanteRNA (Stealth Labels Biotech) and purchased from GenScript as a linearised plasmid. The plasmid had a size of 4370 bp and included the following features: T7 promoter, Kozak sequence, mCherry open reading frame (711 bp), and 5′ and 3′ untranslated regions (UTRs; 32 bp and 111 bp, respectively) optimised for efficient translation. The template was designed to yield an 884 nt long RNA with base composition: U: 15.4%, G: 29.8%, C: 32.7%, A: 22.2% upon running IVT with a T7 RNA polymerase. The distribution of A-stretches in the RNA sequence was: AAAA: 0, AAA: 2, AA: 38, A: 114, and the distribution of C stretches: CCCCCCC: 0, CCCCCC: 1, CCCCC: 2, CCCC: 12, CCC: 10, CC: 40, C: 115.

### Fluorescent nucleoside triphosphates

2CNqATP was synthesized as described in Nilsson *et al.* 2023 (51). The compound was dissolved in RNase-free water and the concentration was determined spectroscopically using Beer-Lambert law (${Ɛ}_{2CNqA}\left(260\;\text{nm}\right)$ = 14,600 M^−1^ cm^−1^ (22)). Aliquots of this stock were stored at −20 °C Cy5-CTP (1 mM) with a propargylamino linker (Jena Bioscience) was acquired and used without further purification.

### IVT and RNA purification

RNase-free consumables were used throughout the work, and prior to handling IVT reagents or RNA, surfaces and equipment were treated with RNaseZap (Invitrogen). IVT reactions were performed using a HighYield T7 RNA Synthesis Kit (Jena Bioscience) with minor adjustments to the supplier’s recommendations. Each reaction was prepared in a total volume of 20 μl containing a Hepes-based reaction buffer (1x), T7 RNA polymerase, dithiothreitol (10 mM), DNA template (50 ng/μl, *vide supra*), and ribonucleoside triphosphates (NTPs). For the 2CNqA IVT reactions, the NTP concentrations were: [UTP] = [GTP] = [CTP] = 7.5 mM, and [ATP + 2CNqATP] = 7.5 mM, with varying fractions 2CNqATP in the adenine pool. For the Cy5-IVT reactions, the NTP concentrations were: [UTP] = [GTP] = [ATP] = 7.5 mM, and [CTP + Cy5-CTP] = 0.3 mM, with varying fractions Cy5-CTP in the cytosine pool. Reactions were run at 37 °C for 4 to 16 h without agitation, whereafter TURBO DNase (0.1 U/μl; Thermo Fisher Scientific) was added and incubated at 37 °C for 15 min to digest the DNA template. The RNA was purified using a RNeasy Spin Column Kit (Qiagen) with minor adjustments to the supplier’s recommendations. The RNA was eluted in RNase-free water and stored in −80 °C.

### Capping and tailing

For translation investigations in cells, the RNA was capped post-transcriptionally using the Vaccinia Capping System (New England Biolabs), installing a Cap0 structure, and polyadenylated using an *Escherichia. coli* poly(A) polymerase (New England Biolabs). Reactions were run according to the supplier’s recommendations. Briefly: prior to capping, the RNA was brought to 65 to 70 °C for 15 min, and then immediately placed on ice for 5 min to facilitate access to the 3′ end of the RNA. Each reaction was then run in a total volume of 20 μl containing uncapped RNA, capping reaction buffer (1X), GTP (0.50 mM), S-adenosylmethionine (0.10 mM), and the Vaccinia Capping System (500 U/μl) at 37 °C for 1 h. Without purification, poly(A) polymerase reaction buffer (1X), ATP (1.0 mM), and poly(A) polymerase (250 U/μl) were added directly to the capping reaction mix, whereafter the tailing reaction was run at 37 °C for 1 h. The reactions were stopped by proceeding directly to the spin column purification (*vide supra*).

### General spectroscopy and UV-vis absorption

All 2CNqA-RNA spectroscopic measurements were performed in phosphate buffered saline (140 mM NaCl, 2.7 mM KCl, 8.0 mM Na_2_HPO_4_, 2.0 mM KH_2_PO_4_) at pH 7.4 in quartz cuvettes (Hellma Materials) at room temperature (*ca*. 22 °C) and an RNA concentration of *ca*. 20 ng/μl. Absorption spectra were recorded on a Cary 4000 UV-vis spectrophotometer (Agilent Technologies) with a scan rate of 600 nm/min and integration time of 0.1 s. For Cy5-RNA, the absorption measurements were performed in water at room temperature (*ca*. 22 °C) on a Nanodrop 1000 spectrophotometer (Thermo Fisher Scientific), using a pathlength of 1 mm.

### Incorporation degree calculations

The fraction of fluorescent bases in the purified RNA, Inc.degree, before capping and tailing, was determined by UV-vis spectroscopy and calculated using the Beer-Lambert law according to Equations (1), (2), (3).

(1) $$C_{Fl}=\frac{A_{Fl}\;\left(\lambda \right)}{{Ɛ}_{Fl}\left(\lambda \right)\times l}$$

(2) $$C_{Can}=\frac{A_{Can}\left(260\;nm\right)}{{Ɛ}_{Can}\left(260\;nm\right)\times l}=\frac{A\left(260\;nm\right)-C_{Fl}\times {Ɛ}_{Fl}\left(260\;nm\right)\times l}{{Ɛ}_{Can}\left(260\;nm\right)\times l}$$

(3) $$Inc.\;degree=\frac{C_{Fl}}{C_{Fl}+C_{Can}}$$

The concentration of fluorescent bases, $C_{Fl}$, was calculated using ${Ɛ}_{2CNqA}\left(360\;\text{nm}\right)$ = 9800 M ^−1^ cm^−1^ (50) and ${Ɛ}_{Cy5}\left(650\;\text{nm}\right)$ = 250,000 M^−1^ cm^−1^ for 2CNqA and Cy5 labelled RNA, respectively. The concentration of canonical bases, $C_{Can}$, was calculated using ${Ɛ}_{2CNqA}\left(260\;\text{nm}\right)$ = 14,600 M^−1^ cm^−1^ (22) and ${Ɛ}_{Cy5}\left(260\;\text{nm}\right)$ = 10,000 M^−1^ cm^−1^ for 2CNqA- and Cy5 labelled RNA, respectively, and using the sequence specific molar absorptivity of an average canonical base, ${Ɛ}_{Can}\left(260\;\text{nm}\right)$ = 9678 M^−1^ cm^−1^, which was calculated using the base composition model, including a hypochromicity factor of 0.9, and the per-base molar absorptivities: ${Ɛ}_{U}$ = 9300 M^−1^ cm^−1^, ${Ɛ}_{G}$ = 11,800 M^−1^ cm^−1^, ${Ɛ}_{C}$ = 7400 M^−1^ cm^−1^, ${Ɛ}_{A}$ = 15,300 M^−1^ cm^−1^. Measurements were performed with 0.3 cm optical pathlength.

### Steady-state fluorescence and fluorescence quantum yield determination

Emission spectra were recorded on a Spex Fluorolog 3 spectrofluorimeter (Jobin Yvon Horiba) from 365 to 700 nm (increment 1 nm, slit width 2 nm), exciting at 360 nm (slit width 2 nm), with 0.1 s integration time. The fluorescence quantum yield, $\Phi_{F}$, of incorporated 2CNqA was determined using quinine sulphate (Sigma Aldrich) in 0.5 M sulfuric acid, $\Phi_{F,ref}$ = 0.54 (59) as reference compound, and calculated according to Equation 4.

(4) $$\Phi_{F}=\Phi_{F,ref}\times \frac{A_{ref}\;}{A_{sample}}\times \frac{\int I_{sample}\;d\lambda }{\int I_{ref}\;d\lambda }\times \frac{\eta_{sample}}{\eta_{ref}}$$

The steady-state fluorescence intensities of the sample ($I_{sample}$) and reference ($I_{ref}$) were integrated from λ = 365 to 700 nm. The solvents’ refractive indices for the sample ($\eta_{sample}$, phosphate buffered saline) and reference ($\eta_{ref}$, 0.5 M H_2_SO_4_) were 1.33 and 1.34, respectively (59). Absorption at the excitation wavelength for the sample ($A_{sample}$) and reference ($A_{ref}$) were adjusted to be below 0.05, to avoid inner filter effects. Quantum yields were determined once per sample. Measurements were performed with 0.3 cm optical pathlength.

### Time-resolved fluorescence

Fluorescence lifetimes ($\tau_{F}$) of the 2CNqA-labelled RNA were measured in a 0.3 cm optical pathlength cuvette using time-correlated single-photon counting, exciting the samples with a 377 nm diode laser (PicoQuant, model LDH-P-C-375,) with a full width at half maximum of 1 nm. A repetition frequency of 10 MHz was provided by a PDL driver (PicoQuant, model 800-B). Emission monochromator was set to 450 nm with a 20 nm spectral bandwidth and the emission polarizer was set to magic angle detection (54.9° with respect to excitation polarization). Photons were collected using a microchannel-plate photomultiplier tube (Hamamatsu, model R3809U with 50 microchannels) and the signal was fed into a LifeSpec II multichannel analyser (Edinburgh Analytical Instruments) using 2048 active channels (24.4 ps/channel) with stop condition of 10,000 photon counts in the top channel. An instrument response function was obtained by measuring the scattering of 0.01 mass-% colloidal silica in water. instrument response function-deconvoluted triexponential functions were fitted to the data points in EasyTau2 (PicoQuant) and amplitude-weighted average fluorescence lifetimes, ⟨τ⟩, were determined according to Equation 5, in which $\alpha_{n}$ is the amplitude of the *n*th component, and $\tau_{n}$ its lifetime.

(5) $$\left\langle \tau \right\rangle =\frac{\Sigma \alpha_{n}\tau_{n}}{\Sigma \alpha_{n}}$$

### Circular dichroism

CD spectra were recorded from 200 to 300 nm on a Chirascan CD spectrophotometer (Applied Photophysics) with 0.1 s integration time, 1 nm steps and 1 nm bandwidth. Samples were measured at RNA concentration ranging from 17 to 25 ng/μl at 1 cm optical pathlength.

### Agarose gel electrophoresis

RNA size was investigated by gel electrophoresis using a gel composed of 1%–2% agarose and SYBRSafe (1X, Thermo Fisher Scientific) in TBE buffer (100 mM Tris, 90 mM boric acid, 1 mM EDTA, Invitrogen), and an identical TBE running buffer. Prior to loading the gel, the RNA samples and RiboRuler High Range RNA Ladder (Thermo Fisher Scientific) were mixed with 2X RNA loading dye (1X final concentration, Thermo Fisher Scientific), whereafter the RNA and ladder were heated to 65 to 70 °C for 5 to 10 min, then immediately placed on ice. The samples (200–300 ng in 10 μl per well) and ladder (4–5 μl per well) were loaded, and the gel was run at constant voltage (10 V/cm, 120–170 mA) for *ca.* 1 h. The gel was imaged on a UV transilluminator (ChemiDoc Touch, BioRad) exciting at 302 nm, with a 590/11 nm emission filter.

### Cell lines and cell culture conditions

Wild type Huh-7 cells were cultured at 37 °C and 5% CO_2_ in Dulbecco’s modified Eagle medium (DMEM GlutaMax, Gibco 21885-025) supplemented with 10% fetal bovine serum (Gibco 10270-106, origin). For sub-cultivation, the adherent cells were washed with calcium- and magnesium-free Dulbecco's phosphate buffered saline (DPBS) (Gibco 14190-144), and detached with 0.25% trypsin-EDTA (Gibco, 25200-056). All cells were cultured in a *mycoplasma* free lab and verified accordingly. For seeding, the cells were detached, counted (after trypsin neutralization), and diluted to 1.5 × 10^5^ cells/ml. For microscopy, four-compartment glass bottom dishes were used (CELLview Dish, Greiner Bio-One) with 450 μl of the cell suspension added per compartment (*i.e.,* 6.8 × 10^4^ cells per compartment). Flow cytometry was performed using 48 flat-bottom well plates (Costar 3548) were 250 μl of the cell suspension was added per well (*i.e.,* 3.8 × 10^4^ cells per well). After seeding, the cells were left to adhere at 37 °C with 5% CO_2_ for 24 h prior to the experiment.

### mRNA transfection

Lipofectamine MessengerMax transfection reagent (Thermo Fisher Scientific) was used according to the supplier’s recommendations. Briefly, lipofectamine, OptiMEM (Gibco, 11058-021), and capped and tailed RNA (2 μl lipofectamine per μg RNA) were combined, mixed, and incubated at room temperature (*ca.* 22 °C) for 5 min. For microscopy, the cell culture medium (CCM) was replaced by a CCM solution containing the lipoplexed RNA (1.5 ng/μl, that is 400 ng RNA per compartment). For flow cytometry, the lipoplexed RNA was added directly to the CCM on the adhered cells (final concentration: 0.8 ng/μl, that is 400 ng RNA per well) followed by a gentle swirl of the plate for mixing. The cells were then incubated at 37 °C with 5% CO_2_ for 24 h prior to analysis.

### Confocal laser scanning microscopy

Microscopy images were captured using a Nikon eclipse T*i*-E microscope with a Nikon C2plus scanner, two parallel Gallium arsenide phosphide detectors, and an Apo 60 × 1.4 Oil λS DIC N2 objective. 2CNqA was excited using a 405 nm laser with emission collected between 410 to 490 nm. mCherry was excited using a 560 nm laser with emission collected between 589 to 720 nm. The signal was optimized for each channel by adjusting the laser power and detector gain. During imaging, the cells were kept in a stage top incubation chamber (OKO lab) maintaining 37 °C and 5% CO_2_. The images were adjusted using ImageJ (https://imagej.net/ij/) for visibility by changing contrast and brightness. Note that imaging data were not used for quantitative analysis in this work.

### Flow cytometry

Following RNA exposure (*vide supra*), the cells were washed twice with DPBS and detached by addition of 0.25% trypsin-EDTA and incubation at 37 °C and 5% CO_2_ for 15 min. The trypsin was neutralized by adding DPBS with 2% FBS whereafter the cells were dispersed by repeated pipetting and transferred to a 96 U-bottom well plate (Corning Costar).

Measurements were performed on a Luminex CellStream flow cytometer operated in autosampler (plate) mode. To promote good cell dispersion and to disrupt eventual clusters, a pre-injection mixing step was employed in the instrument. Samples were probed for mCherry using 560 nm laser excitation with the emission passing through a 611/31 nm bandpass filter. Aspect ratio *versus* forward scattering data of untreated cells was used to define a gating criterion for the single living cell population, from which 4000 to 6000 events per sample were analyzed. The fraction of mCherry-positive cells in the single living cell population was determined based on an intensity cut-off value that was defined using an untreated cell sample (representing the autofluorescence level, see Figs. S8 and S11). The median mCherry intensity in the single living cell population was taken from each intensity histogram, with no further gating. Each exposure condition was run in duplicates on three independent occasions (in total six replicates), and the data is presented as mean ± standard deviation.

### Statistical analysis of fluorophore incorporation as a function of labelling degree

The probability that a base-specific position (*e.g.,* an adenine position) in the sequence is occupied by an analogous modified base (*e.g.,* the adenine analogue 2CNqA) can be calculated by assuming a binominal distribution. If n is the number of base-specific positions in the RNA strand, N the total number of bases, and Inc.degree the average number of modified bases per RNA strand divided by the total number of bases per strand, then the probability, p, that a base-specific position is occupied by an analogous modified base is given by Equation 6.

(6) $$p=Inc.\;degree\times \frac{N}{n}$$

The probability, P(k,n,p), that a randomly selected RNA strand contains exactly k modified bases is given by Equation 7.

(7) $$P\left(k,n,p\right)=\frac{n!}{k!\times \left(n-k\right)!}\times p^{k}\times {\left(1-p\right)}^{\left(n-k\right)}$$

For the special case that a randomly selected RNA strand is unlabelled (k=0), the probability P is given by Equation 8.

(8) $$P_{\left(0,n,p\right)}={\left(1-p\right)}^{n}$$

The generality of Equations (6), (7), (8) should be emphasized. If we instead consider a replacement of a cytosine position, as in our Cy5-CTP experiments or our previously studied tC^O^TP, n is instead the number of available cytosine positions, and if the study concerns a different protein, resulting in a different length of the RNA, N should be changed accordingly.

## Data availability

The data underlying this article will be shared on reasonable request to the corresponding author.

## Supporting information

This article contains supporting information.

## Conflict of interest

The authors declare that they have no conflicts of interest with the contents of this article.
