# Supplementary material for: Expanding fluorescent base analogue labelling of long RNA by in vitro transcription
Source: J Biol Chem. 2025 Oct 16;301(12):110825. doi: 10.1016/j.jbc.2025.110825 (PMC12664369; doi:10.1016/j.jbc.2025.110825)
Supplement: Supporting Figures and Table [file mmc1.docx]

**Supplementary Information for**

**Expanding fluorescent base analogue labelling of long RNA by *in vitro* transcription**

Pauline Pfeiffer^1†^, Alma F.E. Karlsson^1†^, Jesper R. Nilsson^2^, L. Marcus Wilhelmsson^1^*

*^1^ Department of Chemistry and Chemical Engineering, Chalmers University of Technology, Kemivägen 10, SE-41296 Gothenburg, Sweden.*

*^2^ LanteRNA (Stealth Labels Biotech AB), c/o Chalmers Ventures AB, Vera Sandbergs allé 8, SE-41296 Gothenburg, Sweden*

^†^*These two authors contributed equally to this work*

** To whom correspondence should be addressed.*

Contents

[Figure S1. Agarose gels of 2CNqA-labeled RNA 2](#_Toc209968460)

[Figure S2. Reaction yields of IVT 3](#_Toc209968461)

[Figure S3. Molar brightness of RNA strands depending on RNA incorporation 4](#_Toc209968462)

[Figure S4. Fluorescence lifetime of 2CNqA-labeled RNA 5](#_Toc209968463)

[Table S1. Fitted parameters of fluorescence lifetime decays 5](#_Toc209968464)

[Table S2. Extended table of fitted parameters 6](#_Toc209968465)

[Figure S5: Circular dichroism of RNA. 8](#_Toc209968466)

[Figure S6: Control Experiments for microscopy readout. 9](#_Toc209968467)

[Figure S7: Exposure to 1.1%-labelled RNA at 30 min. 10](#_Toc209968468)

[Figure S8. Flow cytometry histograms showing translation of 2CNqA-labeled mRNA into mCherry protein 11](#_Toc209968469)

[Figure S9. Probability of having zero fluorescent bases in the RNA, as a function of incorporation degree 12](#_Toc209968470)

[Figure S10. Cy5 IVT-RNA 13](#_Toc209968471)

[Figure S11. Flow cytometry histograms showing translation of Cy5-labeled mRNA into mCherry protein 14](#_Toc209968472)


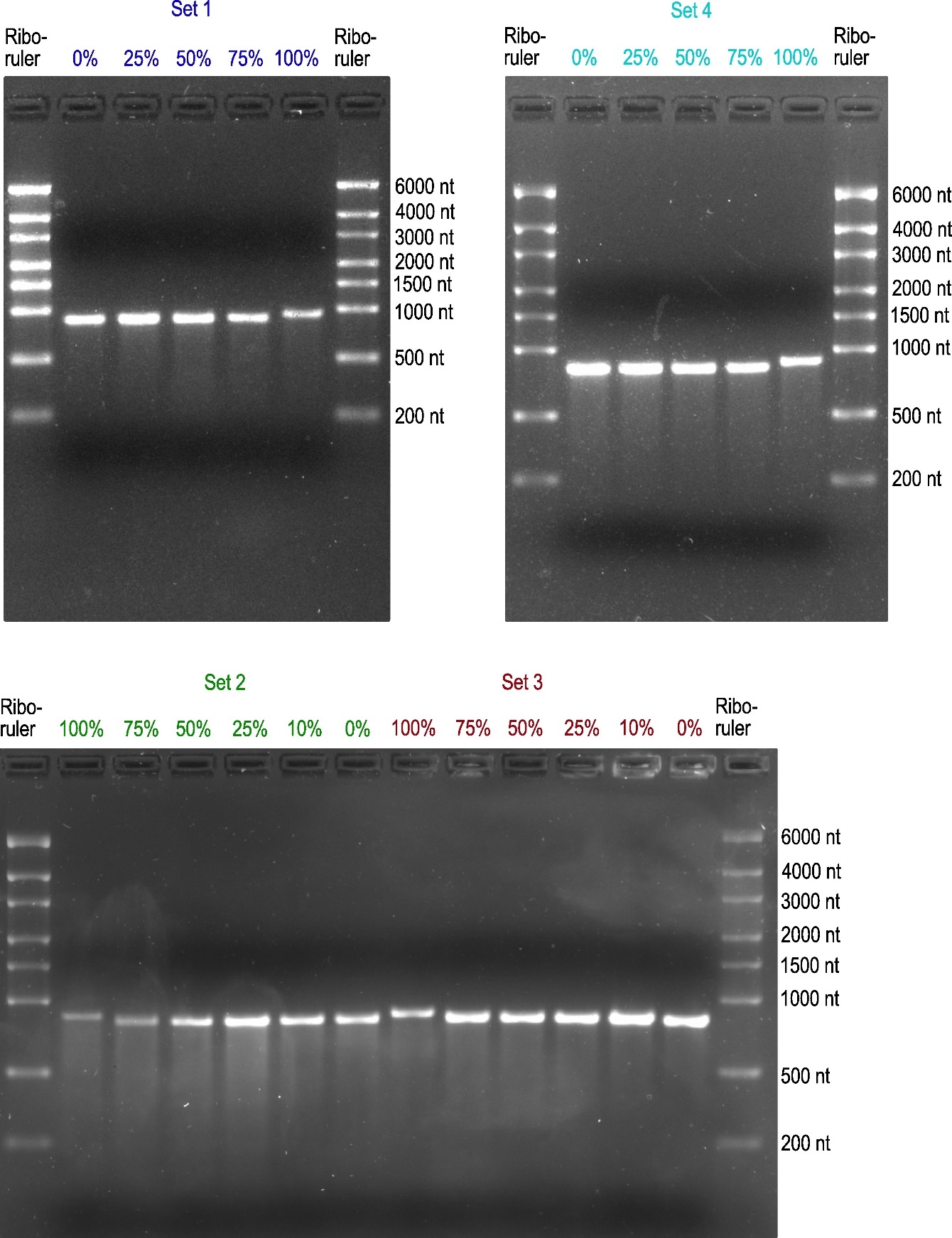

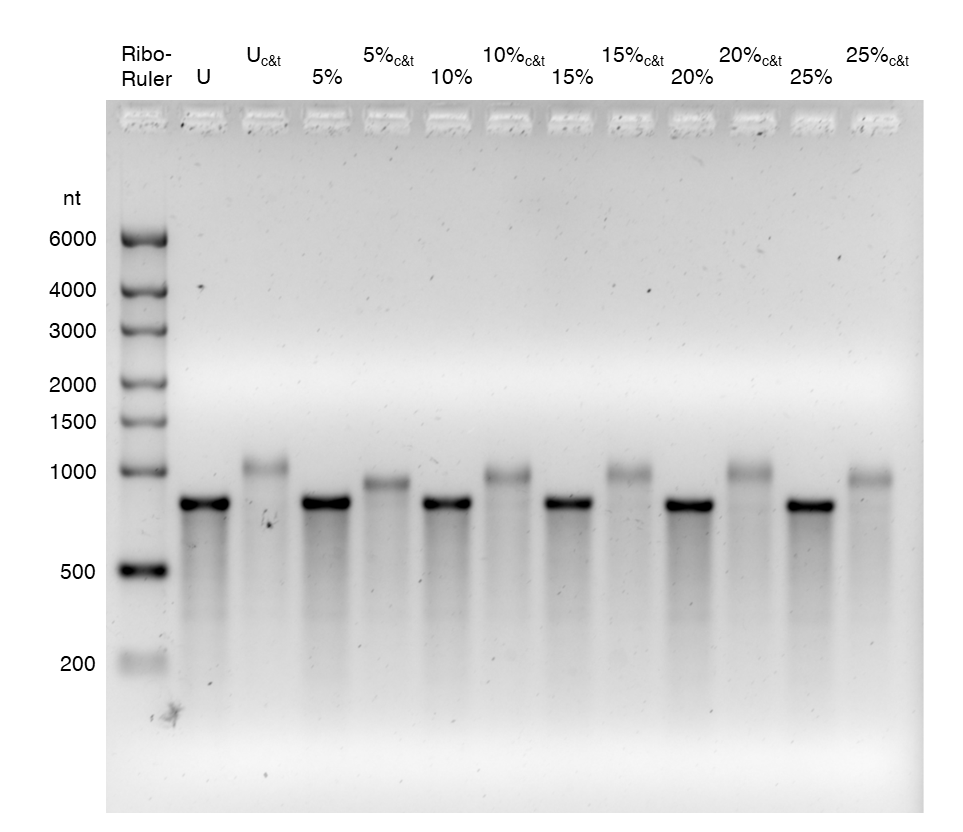


**A**

**B**

Figure S1. Agarose gels of 2CNqA-labeled RNA

**A.** Agarose gel electrophoresis of resulting RNA from four IVT reactions (Set1 – Set4) ran with 0%, 25%, 50%, 75% and 100% 2CNqATP in the ATP-pool. 300 ng RNA per well. **B.** Agarose gel of RNA samples used for lipofection. c&t denotes RNA after the capping and tailing reactions. U: unlabelled RNA. 200 ng RNA per sample well.

Figure S2. Reaction yields of IVT

Reaction yields are expressed as the fraction of maximum amount of RNA that can be produced based on the amount of nucleotide building blocks (NTPs) supplied in the reaction. The theoretical maximum yield is determined by the limiting nucleotide CTP because cytosine has the highest fraction among the nucleotides in the resulting RNA (32.7%, see Experimental Procedures). IVT reactions ran with only natural nucleotides (0%) or with 25%, 50%, 75%, and 100% of the ATP exchanged to 2CNqATP. Data is presented as mean ± standard deviation of four replicates.

Figure S3. Molar brightness of RNA strands depending on RNA incorporation

Molar brightness is calculated as the molar absorptivity of 2CNqA at 260 nm multiplied by the number of 2CNqA per strand and corresponding quantum yield, and accounting for hypochromicity upon base incorporation into RNA.

|  |  |
| --- | --- |
|  |  |

Figure S4. Fluorescence lifetime of 2CNqA-labeled RNA

Fluorescence decays of RNA from one representative IVT series ran with 25%, 50%, 75% and 100% 2CNqATP in the ATP pool (see Experimental Procedures), captured using time-correlated single photon counting. RNA sample data points in dark grey, Instrument response function (IRF) in red, triexponential fits in blue (fitted parameters in Table S1).

Table S1. Fitted parameters of fluorescence lifetime decays

Obtained parameters from fitting deconvoluted multiexponential decays to the fluorescence lifetime decays in EasyTau2. Plotted examples of fits shown in Fig. S4. Each lifetime denoted T_x_ and their amplitudes A_x_. Amplitude averaged fluorescence lifetimes shown as T_AvAmp_ and the chi-square of the fit shown as $x^{2}$. Table continues downwards with all four data sets of RNA from IVT reactions performed with 25%, 50%, 75% and 100% 2CNqATP in the A-pool with the resulting 2CNqA fraction denoted in the table.

| **2CNqA** | **T_1_ (ns)** | **A_1_** | **T_2_ (ns)** | **A_2_** | **T_3_ (ns)** | **A_3_** | **T_AvAmp_ (ns)** | $\boldsymbol{x}^{\mathbf{2}}$ |
| --- | --- | --- | --- | --- | --- | --- | --- | --- |
| **6%** | 10.3 ± 0.1 | 0.39 | 4.3 ± 0.2 | 0.41 | 0.6 ± 0.1 | 0.20 | 5.9 ± 0.1 | 1.19 |
| **11%** | 10.3 ± 0.2 | 0.37 | 4.3 ± 0.3 | 0.42 | 0.7 ± 0.2 | 0.21 | 5.7 ± 0.2 | 1.20 |
| **34%** | 10.1 ± 0.2 | 0.53 | 3.9 ± 0.6 | 0.30 | 0.5 ± 0.2 | 0.18 | 6.6 ± 0.2 | 1.20 |
| **100%** | 11.1 ± 0.2 | 0.18 | 3.5 ± 0.2 | 0.41 | 0.6 ± 0.1 | 0.41 | 3.6 ± 0.1 | 1.35 |
| **8%** | 10.2 ± 0.1 | 0.29 | 4.3 ± 0.2 | 0.43 | 0.7 ± 0.1 | 0.28 | 5.0 ± 0.1 | 1.23 |
| **22%** | 10.2 ± 0.1 | 0.31 | 4.1 ± 0.2 | 0.44 | 0.7 ± 0.1 | 0.25 | 5.1 ± 0.1 | 1.18 |
| **51%** | 10.2 ± 0.1 | 0.28 | 3.7 ± 0.2 | 0.41 | 0.6 ± 0.1 | 0.31 | 4.6 ± 0.1 | 1.28 |
| **106%** | 11.2 ± 0.1 | 0.18 | 3.6 ± 0.1 | 0.40 | 0.6 ± 0.1 | 0.42 | 3.7 ± 0.1 | 1.28 |
| **5%** | 10.4 ± 0.4 | 0.60 | 4.5 ± 0.6 | 0.08 | 0.7 ± 0.4 | 0.32 | 6.0 ± 0.2 | 1.22 |
| **12%** | 10.6 ± 0.2 | 0.35 | 4.8 ± 0.2 | 0.44 | 0.9 ± 0.1 | 0.21 | 6.0 ± 0.1 | 1.09 |
| **35%** | 10.6 ± 0.2 | 0.28 | 4.3 ± 0.3 | 0.45 | 0.8 ± 0.1 | 0.27 | 5.1 ± 0.1 | 1.10 |
| **95%** | 11.6 ± 0.2 | 0.18 | 3.8 ± 0.2 | 0.41 | 0.7 ± 0.1 | 0.41 | 3.9 ± 0.1 | 1.34 |
| **7%** | 10.9 ± 0.2 | 0.56 | 4.8 ± 0.4 | 0.01 | 0.6 ± 0.2 | 0.43 | 5.7 ± 0.2 | 1.36 |
| **14%** | 10.9 ± 0.3 | 0.29 | 4.5 ± 0.5 | 0.44 | 0.6 ± 0.3 | 0.26 | 5.3 ± 0.3 | 1.38 |
| **24%** | 10.6 ± 0.1 | 0.25 | 4.2 ± 0.2 | 0.44 | 0.6 ± 0.1 | 0.31 | 4.7 ± 0.1 | 1.26 |
| **96%** | 11.5 ± 0.4 | 0.12 | 3.7 ± 0.4 | 0.39 | 0.6 ± 0.2 | 0.49 | 3.2 ± 0.3 | 1.60 |

Table S2. Extended table of fitted parameters

Detailed table containing more fitted parameters obtained from fitting of fluorescence lifetime decays in EasyTau2, showing the contributions of each lifetime as raw amplitudes (A_x_) and intensities (I_x_). The shift in instrument response function (Shift_IRF_) and the subtracted backgrounds of the IRF (Bkgr_IRF_) and total signal (Bkgr_Dec_) are also shown. The fraction 2CNqATP in the A-pool in the IVT reaction is denoted at the top and the table continues downward in the same order as the data sets shown in Table S1.

| **Parameter** | **25%** | **50%** | **75%** | **100%** |
| --- | --- | --- | --- | --- |
| **A_1_[kCnts/Chnl]** | 4.6 ± 0.2 | 4.2 ± 0.3 | 6.1 ± 0.5 | 2.1 ± 0.1 |
| **τ_1_[ns]** | 10.3 ± 0.1 | 10.3 ± 0.2 | 10.1 ± 0.2 | 11.1 ± 0.2 |
| **I_1_[kCnts]** | 1 935 ± 58.0 | 1 771 ± 90.0 | 2 530 ± 140.0 | 932.0 ± 36.0 |
| **A_2_[kCnts/Chnl]** | 4.8 ± 0.1 | 4.8 ± 0.1 | 3.4 ± 0.2 | 4.8 ± 0.2 |
| **τ_2_[ns]** | 4.3 ± 0.2 | 4.3 ± 0.3 | 3.9 ± 0.6 | 3.5 ± 0.2 |
| **I_2_[kCnts]** | 842.0 ± 46.0 | 851.0 ± 73.0 | 560.0 ± 130.0 | 686.0 ± 18.0 |
| **A_3_[kCnts/Chnl]** | 2.3 ± 0.1 | 2.4 ± 0.1 | 2.1 ± 0.1 | 4.8 ± 0.1 |
| **τ_3_[ns]** | 0.6 ± 0.1 | 0.7 ± 0.2 | 0.5 ± 0.2 | 0.6 ± 0.1 |
| **I_3_[kCnts]** | 56.0 ± 11.0 | 68.0 ± 17.0 | 39.0 ± 19.0 | 115.0 ± 17.0 |
| **Bkgr_Dec_[kCnts]** | 0.0 ± 0.0 | 0.0 ± 0.0 | 0.0 ± 0.0 | 0.0 ± 0.0 |
| **Bkgr_IRF_[Cnts/Chnl]** | -0.2 ± 0.3 | -0.5 ± 0.2 | 0.1 ± 0.2 | -0.3 ± 0.5 |
| **Shift_IRF_[ps]** | -1.5 ± 0.7 | 5.0 ± 2.8 | 15.3 ± 1.5 | 9.3 ± 2.5 |
| **τ_AvAmp_[ns]** | 5.9 ± 0.1 | 5.7 ± 0.2 | 6.6 ± 0.2 | 3.6 ± 0.1 |
| **A_1_[kCnts/Chnl]** | 3.4 ± 0.2 | 3.6 ± 0.2 | 3.3 ± 0.2 | 2.1 ± 0.1 |
| **τ_1_[ns]** | 10.2 ± 0.1 | 10.2 ± 0.1 | 10.2 ± 0.1 | 11.2 ± 0.1 |
| **I_1_[kCnts]** | 1416.0 ± 46.0 | 1 482 ± 45.0 | 1 386 ± 43.0 | 960.0 ± 27.0 |
| **A_2_[kCnts/Chnl]** | 5.0 ± 0.1 | 5.0 ± 0.1 | 4.8 ± 0.1 | 4.6 ± 0.1 |
| **τ_2_[ns]** | 4.3 ± 0.2 | 4.1 ± 0.2 | 3.7 ± 0.2 | 3.6 ± 0.1 |
| **I_2_[kCnts]** | 870.0 ± 35.0 | 828.0 ± 34.0 | 730.0 ± 34.0 | 679.0 ± 13.0 |
| **A_3_[kCnts/Chnl]** | 3.2 ± 0.1 | 2.9 ± 0.1 | 3.6 ± 0.1 | 4.8 ± 0.1 |
| **τ_3_[ns]** | 0.7 ± 0.1 | 0.7 ± 0.1 | 0.6 ± 0.1 | 0.6 ± 0.1 |
| **I_3_[kCnts]** | 95.0 ± 13.0 | 77.0 ± 12.0 | 83.5 ± 9.9 | 124.0 ± 12.0 |
| **Bkgr_Dec_[kCnts]** | 0.0 ± 0.0 | 0.0 ± 0.0 | 0.0 ± 0.0 | 0.0 ± 0.0 |
| **Bkgr_IRF_[Cnts/Chnl]** | -0.4 ± 0.2 | -0.1 ± 0.2 | 0.7 ± 0.2 | -0.3 ± 0.3 |
| **Shift_IRF_[ps]** | -28.5 ± 1.4 | -9.9 ± 1.2 | -7.0 ± 0.9 | -4.0 ± 1.7 |
| **τ_AvAmp_[ns]** | 5.0 ± ±0.077 | 5.1 ± 0.1 | 4.6 ± 0.1 | 3.7 ± 0.1 |
| **A_1_[kCnts/Chnl]** | 4.3 ± 0.5 | 4.0 ± 0.2 | 3.1 ± 0.2 | 2.0 ± 0.1 |
| **τ_1_[ns]** | 10.4 ± 0.4 | 10.6 ± 0.2 | 10.6 ± 0.2 | 11.6 ± 0.2 |
| **I_1_[kCnts]** | 1 830 ± 160.0 | 1 727 ± 62.0 | 1 361 ± 68.0 | 950.0 ± 29.0 |
| **A_2_[kCnts/Chnl]** | 4.9 ± 0.1 | 4.9 ± 0.1 | 5.0 ± 0.0 | 4.6 ± 0.1 |
| **τ_2_[ns]** | 4.5 ± 0.6 | 4.8 ± 0.2 | 4.3 ± 0.3 | 3.8 ± 0.2 |
| **I_2_[kCnts]** | 910.0 ± 140.0 | 957.0 ± 58.0 | 889.0 ± 36.0 | 729.0 ± 15.0 |
| **A_3_[kCnts/Chnl]** | 2.3 ± 0.2 | 2.3 ± 0.2 | 3.1 ± 0.1 | 4.7 ± 0.1 |
| **τ_3_[ns]** | 0.7 ± 0.4 | 0.9 ± 0.1 | 0.8 ± 0.1 | 0.7 ± 0.1 |
| **I_3_[kCnts]** | 66.0 ± 29.0 | 89.0 ± 12.0 | 103.0 ± 16.0 | 136.0 ± 12.0 |
| **Bkgr_Dec_[kCnts]** | 0.0 ± 0.0 | 0.0 ± 0.0 | 0.0 ± 0.0 | 0.0 ± 0.0 |
| **Bkgr_IRF_[Cnts/Chnl]** | -0.2 ± 0.2 | -0.1 ± 0.2 | -0.3 ± 0.2 | 0.1 ± 0.2 |
| **Shift_IRF_[ps]** | -7.9 ± 1.6 | 0.1 ± 0.7 | 1.4 ± 1.2 | 4.6 ± 1.1 |
| **τ_AvAmp_[ns]** | 6.0 ± 0.2 | 6.0 ± 0.1 | 5.1 ± 0.1 | 3.9 ± 0.1 |
| **A_1_[kCnts/Chnl]** | 3.1 ± 0.2 | 3.3 ± 0.3 | 3.9 ± 0.2 | 6.2 ± 0.4 |
| **τ_1_[ns]** | 0.6 ± 0.2 | 0.6 ± 0.3 | 0.6 ± 0.1 | 0.6 ± 0.2 |
| **I_1_[kCnts]** | 37.0 ± 11.0 | 38.0 ± 16.0 | 47.2 ± 6.6 | 77.0 ± 18.0 |
| **A_2_[kCnts/Chnl]** | 5.3 ± 0.1 | 5.5 ± 0.1 | 5.5 ± 0.1 | 4.9 ± 0.4 |
| **τ_2_[ns]** | 4.8 ± 0.4 | 4.5 ± 0.5 | 4.2 ± 0.2 | 3.7 ± 0.4 |
| **I_2_[kCnts]** | 521.0 ± 44.0 | 512.0 ± 49.0 | 475.0 ± 15.0 | 368.4 ± 4.8 |
| **A_3_[kCnts/Chnl]** | 3.9 ± 0.3 | 3.6 ± 0.4 | 3.1 ± 0.2 | 1.6 ± 0.2 |
| **τ_3_[ns]** | 10.9 ± 0.2 | 10.9 ± 0.3 | 10.6 ± 0.1 | 11.5 ± 0.4 |
| **I_3_[kCnts]** | 872.0 ± 51.0 | 806.0 ± 64.0 | 670.0 ± 23.0 | 367.0 ± 27.0 |
| **Bkgr_Dec_[kCnts]** | 0.0 ± 0.0 | 0.0 ± 0.0 | 0.0 ± 0.0 | 0.0 ± 0.0 |
| **Bkgr_IRF_[Cnts/Chnl]** | 0.1 ± 0.3 | 0.5 ± 0.4 | 0.1 ± 0.2 | 0.5 ± 0.4 |
| **Shift_IRF_[ps]** | 131.4 ± 3.1 | 130.8 ± 8.3 | 128.1 ± 3.3 | 120.0 ± 3.8 |
| **τ_AvAmp_[ns]** | 5.7 ± 0.2 | 5.3 ± 0.3 | 4.7 ± 0.1 | 3.2 ± 0.3 |


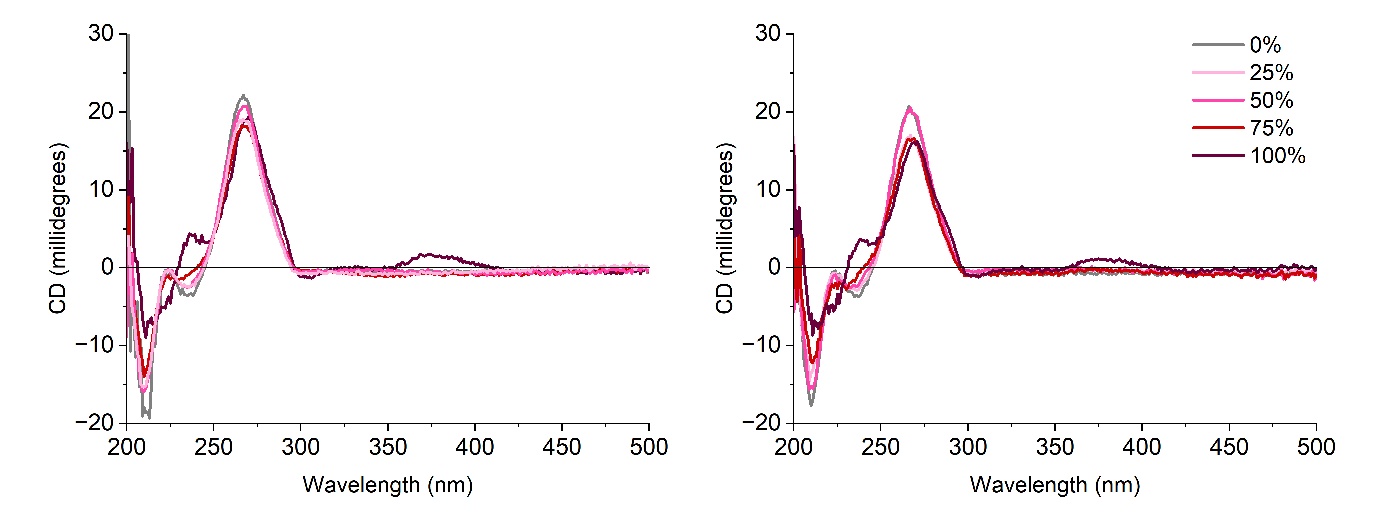


Figure S5: Circular dichroism of RNA.

Circular dichroism of RNA in PBS, measured at 25 ng/µL, from two sets of IVT reactions with 25%, 50%, 75% and 100% 2CNqATP in the A-pool. When all adenines are exchanged to 2CNqA (100% 2CNqATP in the A-pool), a positive CD signal appears at 238 nm and 380 nm. The CD of labelling degrees up to 75% are almost unaltered, with only slight decrease in negative CD at 238 nm, compared to unlabelled RNA. Three isodichroic points are found at 217 nm, 229 nm and 249 nm.

**
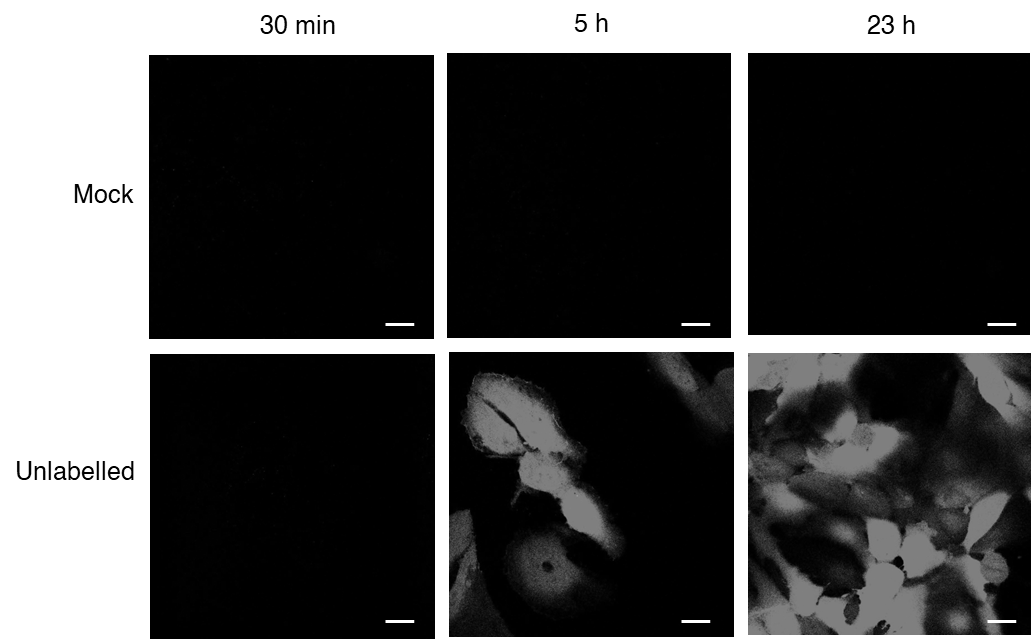
**

Figure S6: Control Experiments for microscopy readout.

Live-cell confocal microscopy of Huh-7 cells exposed to lipofectamine without RNA (Mock) and to unlabelled RNA (0% 2CNqATP). Shown are the images of mCherry channel (λ_ex_ = 560 nm, λ_em_ = 589–750 nm) after the indicated exposure time. Scale bars = 20 µm.

**
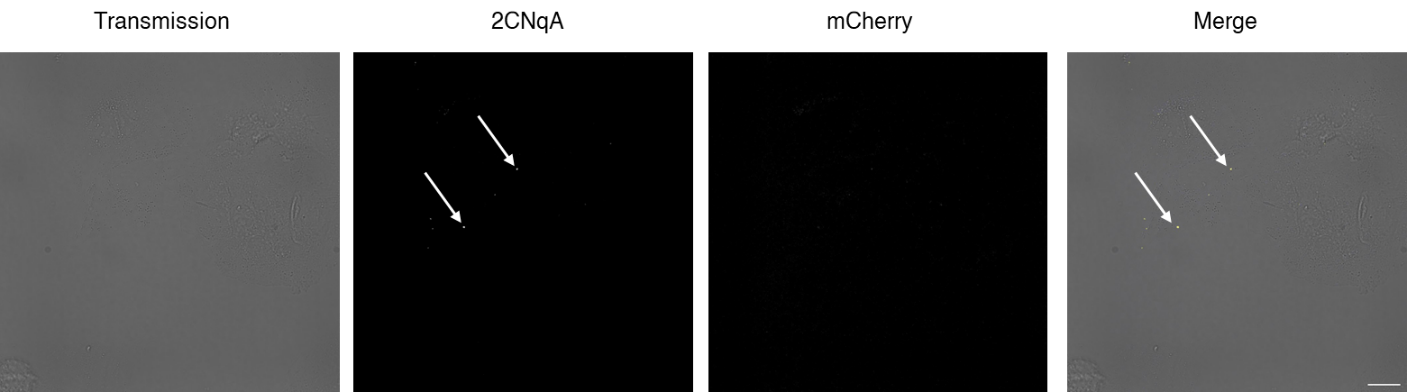
**

Figure S7: Exposure to 1.1%-labelled RNA at 30 min.

Live-cell confocal fluorescence microscopy of Huh-7 cells exposed to 1.1%-labelled 2CNqA-RNA for 30 min. Detection channels are 2CNqA (cyan; λex = 405 nm, λem = 410–490 nm) and mCherry (red; λex = 560 nm, λem = 589–750 nm). Scale bars = 20 µm.


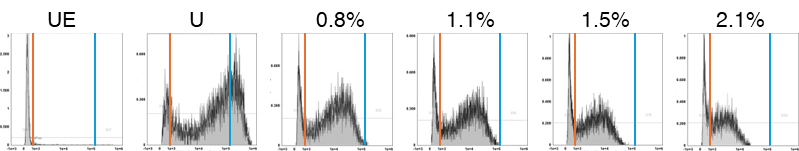


Figure S8. Flow cytometry histograms showing translation of 2CNqA-labeled mRNA into mCherry protein

Flow cytometry analysis of Huh-7 cells exposed to 2CNqA-labeled RNA for 24 h, where only single living cells were included in the analysis (see Experimental Procedures). Representative histograms of the fluorescence intensity recorded in the mCherry channel are shown. Orange and blue lines were placed at the same intensities in all histograms, with the orange line indicating gated autofluorescence level, meaning mCherry positive level to the right (UE = unexposed cells), and the blue line indicating the median of mCherry synthesis from unlabelled mRNA (U). The fractions on top of the histograms indicate 2CNqA incorporation degree in the RNA, *i.e.* the percentage 2CNqA of all bases, excluding the poly(A) tail.

Figure S9. Probability of having zero fluorescent bases in the RNA, as a function of incorporation degree

A binomial distribution was assumed to calculate the probability that a randomly selected RNA strand is unlabelled (see equation 8 in Experimental Procedures), given the indicated number of cytosine or adenine positions, and total number of nucleotides, in the RNA. Note that the two lines overlap almost entirely, with the red line covering the black.





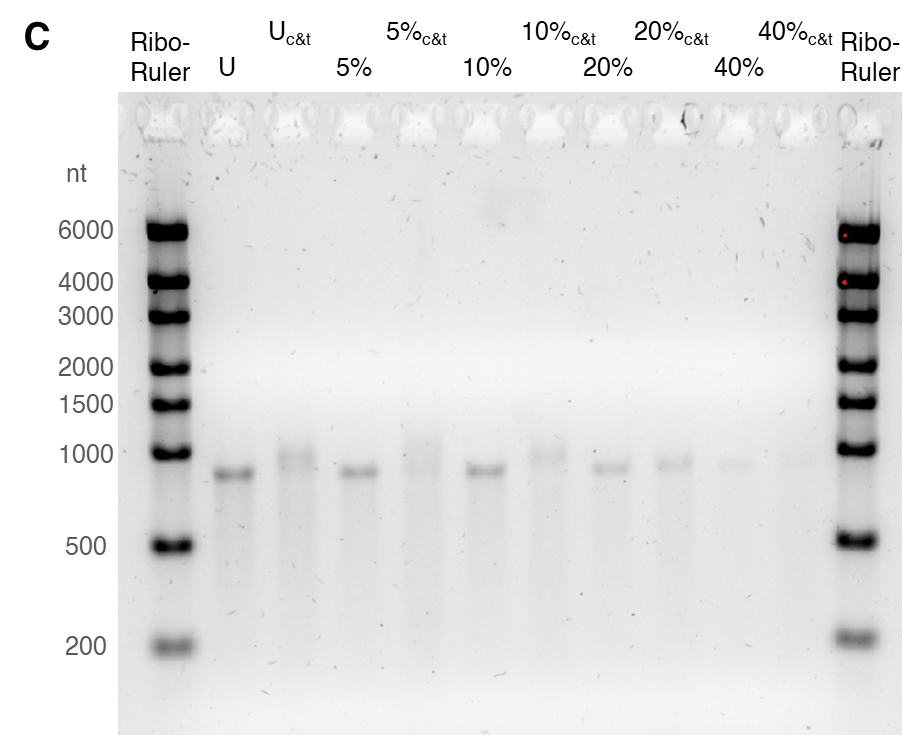


Figure S10. Cy5 IVT-RNA

**A.** UV-vis absorption spectra of purified Cy5 IVT-RNA from five reactions ran with 0%, 5%, 10%, 20%, and 40% fraction of Cy5-CTP in the CTP pool (light to dark colour). **B.** Analysis of incorporation of IVT reactions containing Cy5-CTP (brown), compared to reactions containing 2CNqATP (light grey, identical data to Fig. 2), and a line with slope = 1 as reference (grey dashed line). Left y-axis applies to incorporation of both dyes and the reference slope, while the y-axis on the right applies only to the incorporation of Cy5 in brown. **C.** Agarose gel electrophoresis of the purified Cy5 RNA prior to and after capping and tailing reaction (c&t). Note that the SYBRSafe emission is quenched upon interaction with the Cy5 in the RNA, and that this effect is more pronounced as the Cy5 incorporation degree increases.


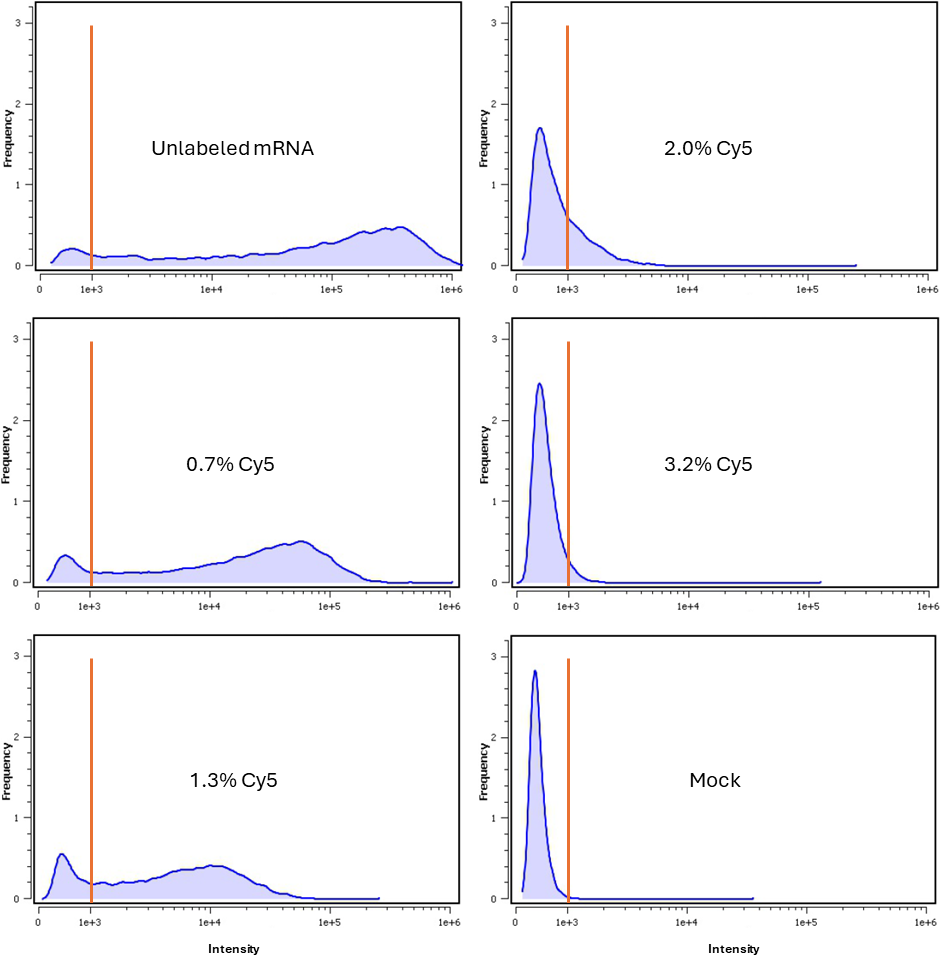


Figure S11. Flow cytometry histograms showing translation of Cy5-labeled mRNA into mCherry protein

Flow cytometry analysis of Huh-7 cells exposed to Cy5-labeled RNA for 24 h, where only single living cells were included in the analysis (see Experimental Procedures). Representative histograms of the fluorescence intensity recorded in the mCherry channel are shown. The fraction mCherry-positive cells as presented in Fig. 4 was determined by setting a bisector gating based on the Mock exposure histogram (orange line). The fractions in the histograms indicate the Cy5 incorporation degree in the RNA, *i.e.* the percentage Cy5 of all bases, excluding the poly(A) tail.
